# Compiling Data of Workforce Policies: A Data Compendium About Direct Care Workers Caring for People With Dementia

**DOI:** 10.1016/j.jamda.2025.105939

**Published:** 2025-10-26

**Authors:** Lei Chen, Kyoko Peterson, Joanne Spetz

**Affiliations:** a Philip R. Lee Institute for Health Policy Studies, University of California San Francisco, San Francisco, CA, USA; b Healthforce Center at UCSF, University of California San Francisco, San Francisco, CA, USA

**Keywords:** Direct care workers, workforce, datasets, policy, long-term care

## Abstract

Direct care workers in long-term care settings are essential to provide care for older adults and people with disabilities who have daily needs. There is an increasing demand for an adequately sized and well-prepared direct care workforce to support people living with dementia due to its rising prevalence. However, a lack of standardized policies and data leaves researchers and policymakers with little evidence to guide the development and refinement of workforce policies. The National Institute on Aging—funded Advancing Workforce Analysis and Research for Dementia Network is developing a compendium of policies and programs related to the demand, supply, working conditions, and skills of direct care workers, including dementia care—related attributes. For each policy, the compendium provides a description of the policy, an annotated list of related datasets, and a downloadable harmonized dataset with research-ready data. The data compendium is innovative in its organization of data and resources by policy topic. It encompasses a diverse range of sources, including government reports, organizational resources, large datasets, and data collected by researchers, and it can be linked with other data sources. The creation of a crowd-sourced repository of data on state and federal regulations in the United States will facilitate the rapid advancement of research and development for early stage researchers. The data compendium identifies research gaps that can be filled by leveraging these data, which have the potential to inform evidence-based policy and program development related to the long-term care workforce.

Demand for direct care workers (DCWs) in long-term care (LTC) settings is increasing due to the aging of the baby boomer population and the rising prevalence of Alzheimer’s disease and related dementias.^1^ DCWs comprise roughly 25% of the LTC workforce and provide 80% of all hands-on care across LTC settings.^2^ The direct care workforce also provides most care for individuals living with dementia.^3^ However, a lack of data and research leaves us with little evidence to guide the development and refinement of policies, despite the growing importance of ensuring an adequately sized and well-prepared direct care workforce.^4,5^ This special article introduces an innovative data compendium that developed a research-ready, harmonized dataset of policies and programs affecting DCWs’ demand, supply, working conditions, and skills.

## Why Do We Need This Data Compendium?

### Challenges That DCWs are Facing

DCWs―including personal care aides, home health aides, and nursing assistants―assist with activities of daily living and instrumental activities of daily living for older adults and people with disabilities across diverse settings.^6^ Job growth for DCWs is expected to be very high over the next decade. The direct care workforce is projected to add 1.3 million new jobs in the United States from 2018 to 2028, including >1 million home care jobs―more new jobs than any other occupation in the country.^4^ However, from 2010 to 2019, there was little growth in the direct care workforce in the United States, despite the growing demand.^7^ DCWs face challenges such as low compensation, physically and emotionally demanding workloads, scheduling difficulties, inadequate supervision, and limited training and career advancement prospects. These challenges contribute to job dissatisfaction and high turnover rates among DCWs.^4,8^ Caring for people with dementia requires strong interpersonal skills and technical knowledge to manage challenging behavioral and psychological symptoms.^4,9^ Additional challenges include a lack of time to provide personalized care; a lack of knowledge about dementia and its symptoms, evolution, and available treatments; difficulty in communicating with patients; and a shortfall of supportive management practices.^10^

### Lack of Policies and Research Data

There are no standardized policies for DCWs in the United States.^11^ The direct care workforce is impacted by a range of policies and programs across states, payers, and health care organizations. This includes differences in training requirements, state-level regulations governing the services DCWs are permitted to provide, and reimbursement strategies that impact the demand for and compensation of DCWs.^11^ For example, although federal regulations apply to a limited subset of the workforce, state-level training standards vary considerably by state, LTC setting, and job title. This variation can make it challenging for DCWs to transfer their skills across settings, such as from home care to residential care, thereby restricting career advancement opportunities and reducing the overall adaptability of the workforce.^11^ Moreover, there is limited evidence on how these policies affect the quality of care received by people living with dementia.^12^

Scholars new to this area of research and policy understandably find it challenging to identify and understand state and federal regulatory and practice structures, creating a barrier to entry into this research field and hindering the development of rigorous research and its translation into practice and policy.^11^ The direct care workforce also encompasses multiple occupations that have overlapping definitions and job descriptions, which can be perplexing for researchers to operationalize for research purposes, posing challenges to the generalizability of research findings.^11^ Federal and state data collection systems remain insufficient and underfunded in their capacity to track and measure critical dimensions of the direct care workforce, including size, stability, credentials, and compensation.^11^ This lack of robust, comprehensive data limits the ability of policymakers, industry leaders, and other LTC stakeholders to make informed decisions aimed at improving job quality and workforce outcomes.^11^ Multiple national panels and research reviews have identified research needs to guide the advancement of the workforce that provides care to people living with dementia.^13–15^

### Advancing Workforce Analysis and Research for Dementia Network Data Compendium

The Advancing Workforce Analysis and Research for Dementia (AWARD) Network (supported by the US National Institute on Aging) is developing a research-ready harmonized dataset of US policies and programs that affect the demand, supply, working conditions, and skills of DCWs. Selected information is compiled into a database, and all data elements are accompanied by a detailed data codebook and manual that explains each variable, its source, its definition, and associated caveats.

## Process and Contents of Developing the Data Compendium

### Process of Organizing Resources and Data

**Fig. 1.**
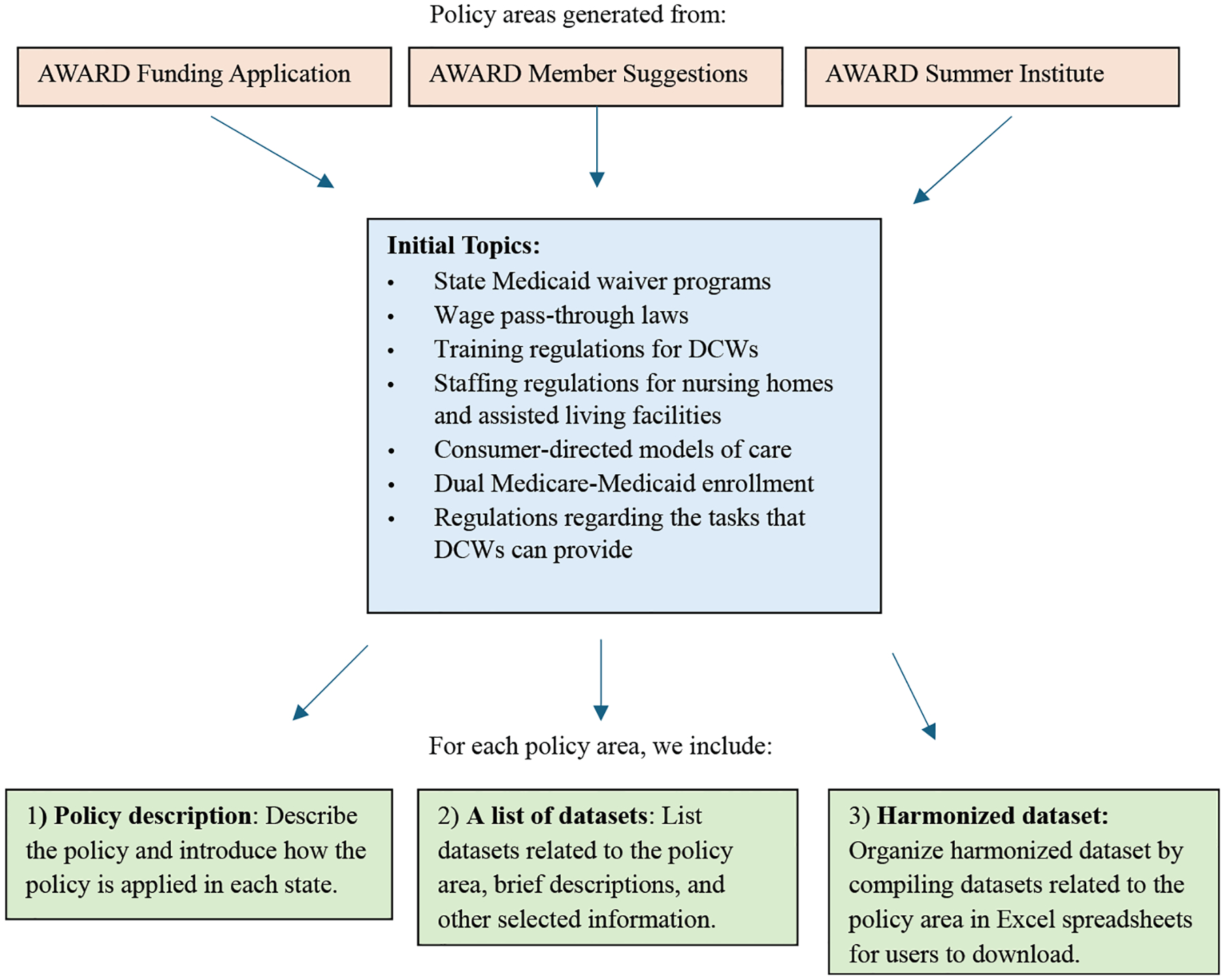
Process of developing the data compendium. We started from selecting 7 policy areas based on 3 resources. For each policy topic, we included 3 components for the data compendium.

We selected policy areas related to DCWs caring for people with dementia based on suggestions from >20 experts on the health workforce that cares for people with dementia, and on suggestions provided by the AWARD Network members at 2 annual meetings and the network’s first Summer Training Institute in 2024. After reviewing the suggestions received, we began with 7 topics: state Medicaid waiver programs, wage pass-through laws, training regulations for DCWs, staffing regulations for nursing homes and assisted living facilities, consumer-directed models of care, dual Medicare-Medicaid enrollment, and regulations regarding the tasks that DCWs can provide. The process of developing the data compendium for each topic is shown in Figure 1. For each policy area, we included the following:

1. A policy description, which refers to descriptions of the general policy area across states and how it was applied in each state based on reports from governments and research studies. The contents of the policy description include an introduction to the policy, its application across states, examples of programs or policies from at least 1 state, empirical studies evaluating the policy’s impact, and a summary highlighting existing research findings and opportunities for future research.
2. A list of datasets, including the time period of data collection, tags related to the topics, type of dataset (public or proprietary), list of related variables, examples of published articles using the dataset, relevant links, limitations of the dataset, and whether the data were included in our harmonized dataset. There are limited datasets directly related to DCWs caring for people with dementia. Our data compendium thus includes datasets that can inform research on the broader health care workforce caring for people in LTC settings. The sources identified in this step included individual-level data that allow researchers to delve more in-depth. The inclusion criteria were the following: (1) data collected within the past 10 years, and (2) data relevant to the policy area that can support related studies.
3. A harmonized dataset that compiled variables from various data resources related to the policy area, provided as Excel (Microsoft Inc) spreadsheets for users to download, including technical documents (list of variables and their information) and research-ready state-level data. The variables included in the harmonized dataset resources were either publicly available or shared with permission from the owners, allowing researchers to use them directly. Inclusion criteria were as follows: (1) data organized at the state level, (2) data collected within the past 10 years, and (3) data relevant to the policy area that can support related studies. We excluded data that were not publicly accessible, not published within the past 10 years, organized at the individual level, or with a significant amount of missing data. After cleaning, datasets were merged by state, and variables were renamed to ensure consistency across the harmonized dataset. Detailed documentation from the original sources was provided to clarify how the data were created.

Here, we use the policy areas of wage pass-through laws and training regulations for DCWs as 2 examples to demonstrate how the resources and data are organized. Detailed information on all topics is presented in Table 1.

### Example 1: Wage Pass-Through Laws

#### Policy description

Inadequate compensation for DCWs, especially when compared with jobs with similar entry requirements, is a long-standing issue in LTC.^19,20^ In the late 1980s, states began implementing laws to address this problem, some of which were described as wage pass-through laws in the late 1990s.^21^ In the policy description of the data compendium, we outlined the issue of inadequate compensation for DCWs, types of wage pass-through laws, details of wage pass-through laws in each state, and summaries of research on the effects of wage pass-through laws on DCWs’ wages.^22^ We also presented a case study to describe the wage pass-through program in Illinois.^21^ The resources were sourced from the Institute of Healing Justice and Equity, the National Governors Association, US Federal Reserve Economic Data, US Assistant Secretary for Planning and Evaluation, and peer-reviewed journal articles related to this policy.^22^ Most academic publications on their impact date back to 10 years ago―perhaps because the first laws were enacted >20 years ago―and there is limited recent research on this topic. However, discussions about improving recruiting to the LTC workforce during and after the COVID-19 pandemic have revived the topic.^22^

#### List of datasets

For wage pass-through laws, we identified 3 data resources that could be relevant for researchers: (1) an online platform that contains facility-level data for nursing homes, including facility-level staffing, facility characteristics, and resident characteristics; (2) a dataset that provides data on nursing homes in the United States and contains a variable indicating whether the facility is subject to a wage pass-through policy (although this resource has not yet been used to research wage pass-through laws); and (3) a survey that provides individual-level and household-level data on nursing home care in the United States that can be used to study employment status, income, insurance coverage, and other information on DCWs. More details of these datasets are provided in the data compendium.^22^

#### Harmonized dataset

The harmonized dataset of the wage pass-through laws is composed of 33 variables related to the design of the law, settings of care to which the law applies, median wages, state-level minimum wages, and differences between DCW median wages vs wages for occupations with similar or low entry-level requirements. These variables were sourced from various places, including data from government reports, organizational resources, and research institutes’ analyses.^22^

### Example 2: Training Regulations for DCWs

#### Policy description

The policy description begins with an introduction to 3 types of DCWs - personal care aides, home health aides, and nursing assistants. It then describes general training requirements and regulations for these roles at the federal and state levels. Training requirements vary widely across roles and institutions.^23^ In addition, the methodology by which state labor agencies classify direct care roles can be unclear.^23^ The lack of training requirements reinforces the misconception that direct care is low-skill work, leaving workers unprepared for complex demands, reducing job satisfaction and retention, and directly impacting the provision of care.^3^ We introduced the direct care training programs in Indiana, Colorado, and Wisconsin as examples of well-developed state policies. For this policy area, we specifically introduced dementia-specific training and considerations guided by the Alzheimer’s Association.^3^ Other resources were derived from PHI and the National Governors’ Association.^23^ Overall, we concluded that training requirements for DCWs are limited and differ significantly by job classification, care setting, and state. Dementia-specific training requirements are even more limited. Researchers have collected data about DCWs’ training by conducting surveys, interviews, interventions, and analyzing state regulations.^24^

#### List of datasets

For the topic of training regulations for DCWs, we identified 3 relevant data resources: (1) a state-by-state analyses of training requirements for personal care aides, home health aides, and nursing assistants; (2) periodically published reports summarizing assisted living licensure or certification requirements, including direct care staff education and dementia-specific training and regulations for all 50 states and the District of Columbia; and (3) a published breakdown of federal and state requirements for dementia training.^24^

#### Harmonized dataset

The harmonized dataset of training regulations for DCWs is composed of 40 variables related to training hours, standards, specifications, contents, orientation requirements, and supervision requirements across different types of DCWs. These variables were compiled from multiple sources, including data from organizations, research institutes, and peer-reviewed publications.^24^

### Innovations of the Data Compendium

#### Innovation 1: Organized Based on Policies and Regulations

Federal regulations govern some programs and policies related to DCWs, whereas other regulatory issues fall under the purview of states, particularly Medicaid programs and regulations governing the health workforce.^25,26^ Most data compendia related to the health care workforce or LTC are either organized by specific datasets or a single policy. For example, the Albany Health Workforce Research Center data compendium is organized by 45 federal data sources that can be used for health workforce analyses, including information about the lead federal agency, website, description of data source, relevance for health workforce analysis, geographic detail, and availability.^27^ Another example is in nursing research, where there have been different and sometimes conflicting measures of scope of practice laws for nurse practitioners, thereby limiting their utility to policymakers. McMichael and Markowitz^28^ developed a coding scheme for nurse practitioner scope of practice laws based on a detailed legal review of state statutes and regulations. Our data compendium is among the first to organize resources and data at the state level according to policies and regulations related to DCWs caring for people with dementia. For each policy topic, we expanded our search criteria to include policies and regulations related to health care workers in various LTC settings (eg, home and community-based settings, nursing homes, assisted living facilities) because specific policies and data regarding DCWs caring for people with dementia are limited. After reviewing relevant policies, reports, and peer-reviewed journal articles, we summarized the ways in which these policies and regulations could impact DCWs caring for people with dementia, and the research and policy gaps that warrant further examination.

#### Innovation 2: Resources and Data Sources for Each Policy Topic

The other innovation is that the AWARD Network data compendium includes multiple resources and datasets. In addition to including large datasets or data platforms that are often used by researchers in academia (eg, Medicaid claims Transformed Medicaid Statistical Information System Analytic Files, Long-Term Care: Facts on Care in the U.S.), we also included government reports (eg, Centers for Medicare & Medicaid Services 372 Reports), organization data and reports (eg, KFF State Health Facts data, PHI data), and researchers’ own data collection (eg, training regulations data collected by Kelly et al^16^). We identified websites that provide information about relevant policies and regulations. We also contacted the authors of publications to request that they share data on regulations and policies that they organized related to a specific topic. For each topic, we included multiple types of data sources, which are important for public health and policy assessment.^29,30^ Given that the definitions of measures and methods of data collection varied across different data sources and time periods, we noted these differences and made the notes as clear as possible.

#### Innovation 3: Potential to Link With Other Data Sources

This data compendium can be linked with other data sources that provide a geographic identifier at the state level. For example, it can be linked to the National Dementia Workforce Survey, the first and largest nationally representative survey of the professional dementia care workforce in the United States.^31^ For National Institute on Aging (NIA)—funded research, linkages can be completed within the NIA LINKAGE program, which connects NIA-funded study data with existing datasets from the Centers for Medicare & Medicaid Services and other sources, and establishes a cloud-based environment to support data accessibility and sharing.^32^ Future plans include adding control variables or outcome variables to the data compendium, which can include states’ characteristics, including demographics, staffing numbers, nursing home retention rates, the number of different types of occupations among DCWs, payment sources for home and community-based service programs, number of people who have dementia, health and well-being status in states, and so forth. The linkage of these data elements will provide opportunities to examine the characteristics and impact of policies and programs related to DCWs on health and workforce outcomes across different states.

## Implications for Policy, Research, and Practice

### Implications for Research

The creation of a crowd-sourced repository of data on state and federal regulations regarding DCWs’ demand, supply, working conditions, and skills will contribute to the rapid advancement of research. The AWARD Network data compendium provides policies in one place and serves as a valuable resource for future research. It organizes ready-to-use data resources for early stage researchers and identifies research gaps that can be filled in by leveraging these data. For example, there is a lack of studies using data related to state Medicaid waiver programs to examine the needs of the direct care workforce, which deserves further examination.^33^ The data compendium innovatively integrates existing resources and data from diverse sources to describe and examine the impact of policies, including government reports, organizational resources, large datasets, and data collected by researchers, which are underused by researchers for policy-related work regarding the LTC workforce. For example, researchers collected data about DCWs’ trainings by conducting surveys, interviews, interventions, or organizing state regulations; however, they did not often use existing data organized by organizations (eg, PHI, National Center for Assistive Living) to examine training regulations for DCWs. Moreover, more studies are needed to understand the relationship between specific staffing regulations for dementia care and care outcomes in assisted living facilities.^34^ The other contributions of this data compendium are not only for DCWs caring for people with dementia, but can also inform the development of future data compendia related to policies and programs about the health care workforce.

### Implications for Policy and Practice

In the policy arena, the AWARD Network data compendium provides data and identifies research gaps that have the potential to inform evidence-based policy and program development, which is crucial for supporting DCWs caring for people with dementia. The data compendium includes policies and programs related to Medicaid, which is a primary payer for the long-term services and supports needs of people living with dementia, and is also especially important at the state level to support DCWs.^35,36^ States have the authority to regulate professions and control Medicaid programs, which pay for more than one-half of long-term services and supports in the United States.^12,25,26^ Medicaid waiver programs―one topic included in the data compendium―allow states to test innovative approaches to Medicaid benefits, including home and community-based services where DCWs are employed.^8^ Wage pass-through laws can direct Medicaid funds to increase DCWs’ compensation through reimbursement mechanisms.^20^ Researchers and policymakers can use the data compendium to examine whether wage pass-through policies are related to improved DCW recruitment, retention, or satisfaction, and assess which waiver structures are more strongly linked with these outcomes. The results can be used to advocate for or refine these programs to better achieve their intended goals of supporting workforce stability and care quality. The compendium organizes data on staffing regulations and training for nursing homes and assisted living facilities, highlighting the importance of staffing levels and training on care outcomes.^34^ Thus, the data can be used to develop targeted programs and state guidance to optimize staffing ratios, qualifications, and training requirements for DCWs in dementia care settings.

### Limitations, Challenges, and Future Directions

The value of the data compendium highlights the crucial role of expanding research about DCWs in LTC settings, particularly as greater numbers of LTC residents and clients live with dementia. Development and maintenance of the data compendium is an ongoing process that will be updated in response to policy changes and new evidence as they emerge over time. However, several limitations and challenges should be acknowledged. First, the compendium reflects the state of policies at specific points in time, and real-time updates are not feasible with the staff resources of the AWARD Network. Although a dataon column specifies the date when each data point was organized, some original sources continue to update their data, potentially leading to temporal variability of the data. Second, limited empirical studies exist regarding the impact of state and federal policies on DCWs, especially those caring for people with dementia. As a result, the compendium may not capture all relevant nuances or emerging policy effects.

The harmonized dataset will be updated in the final year of the funded project. Future iterations of this work will prioritize data more specifically related to DCWs who care for people with dementia to better inform policy and program development in this area.^37^ Exploratory interviews with DCWs caring for people with dementia may help identify new policy topics for future inclusion.

Dissemination of the compendium to relevant stakeholders is ongoing. The data have been presented at 2 national conferences and during the Summer Training Institute, and continued outreach through conferences, webinars, and other channels is needed to ensure broad awareness and uptake.

## Figures and Tables

**Table 1:** Detailed Information and Sources on All Topics of the Data Compendium

Topic	Policy Description Contents	List of Datasets	Harmonized Dataset Sources
State Medicaid Waiver Programs	Types of Medicaid waiver programs HCBS Community Care Network for Dementia	T-MSIS TAF Medicaid Analytic eXtract CMS 372 Reports KFF Annual HCBS Survey	KFF Urban Institute CMS-64 report CMS-372 report Medicaid and CHIP Scorecard
Wage pass-through laws	Inadequate compensation for DCWs Types of WPTs Details of WPTs in each state Effect of WPTs in each state Effects of WPTs on DCWs wages	OSCAR CASPER LTCFocus SIPP	Institute of Healing Justice and Equity National Governors Association Federal Reserve Economic Data Assistant Secretary for Planning and Evaluation KFF
Training regulations for DCWs	Types of DCWs Variations in DCWs training Inadequate training Ways to improve training Dementia specific-training and consideration	PHI data Assisted Living State Regulatory Review National Council of Certified Dementia Practitioners	PHI RTI Data collected by Kelly et al^16^
Staffing regulations for nursing homes and assisted living facilities	Services that nursing homes and assisted living facilities provide Current staffing regulations Importance of staffing regulations on improving care quality Number of older adults with dementias in these settings Specific staffing regulations for caring for people with dementia	Consumer Voice National Center for Assisted Living The Medicaid and CHIP Payment and Access Commission Payroll Based Journal Daily Nurse Staffing	KFF Data organized by Kaskie et al (2015)^17^ RTI
Dual Medicare-Medicaid enrollees	Explanation of Medicare Explanation of Medicaid Who are dual-eligibles Enrollment patterns and spending patterns for dual-eligibles	KFF State Health Facts Data	KFF
Consumer-directed models of care	Introduction of consumer-directed models Consumer-directed models and DCWs Consumer-directed models in each state	KFF Medicaid State Plan Personal Care Program Survey ACL Veteran-Directed Care Program	KFF Tyler et al (2024)^18^ American Council on Aging AARP LTSS Scorecard
Regulations regarding the tasks that direct care workers can provide	Nurse delegation Scope of practice regulations in California Impact on care	AARP LTSS Scorecard	AARP UCSF Health force Center report

ACL, Administration for Community Living; AARP, American Association of Retired Persons; CMS, Centers for Medicare & Medicaid Services; CHIP, Children’s Health Insurance Program; CASPER, Certification and Survey Provider Enhanced Reporting; DCWs, direct care workers; HCBS, home- and community-based services; LTCFocus, Long-Term Care: Facts on Care in the U.S.; LTSS, Long-term Services and Support; OSCAR, The Online Survey Certification and Reporting; RTI, Research Triangle Institute; SIPP, the Survey of Income and Program Participation; T-MSIS TAF, Transformed Medicaid Statistical Information System Analytic Files; UCSF, University of California San Francisco; WPTs, wage pass-through laws.
